# Supplementary material for: Complexome profiling of the Chlamydomonas psb28 mutant reveals TEF5 as an early PSII assembly factor
Source: Plant Cell. 2025 Mar 17;37(6):koaf055. doi: 10.1093/plcell/koaf055 (PMC12164590; doi:10.1093/plcell/koaf055)
Supplement: koaf055_Supplementary_Data [file koaf055_supplementary_data.zip › psb28_interactive.zip!/plots/intensity/qualityPlots/peptides/RB60_Cre02.g088200.t1.2.html]

### Cre02.g088200.t1.2

Trivial name: RB60  
SeqCoverage [median]: 55.60  
Mass: 58.24 kDa  
  
 

| Sequence | MissedCleavage | Mass [Da] | wtIntensity | muIntensity | Score |
| --- | --- | --- | --- | --- | --- |
| AAAPDALIAK | 0 | 939.54 | 8773.44 | 17762.08 | 107.69 |
| AAAPDALIAKVDATQEESLAQK | 1 | 2239.17 | 0.00 | 1887.78 | 92.10 |
| AAGLDAVDTVSVVK | 0 | 1343.73 | 21667.46 | 42615.63 | 158.05 |
| AATALKAAAPDALIAK | 1 | 1494.88 | 0.00 | 522.62 | 33.30 |
| ADAEIMTVFR | 0 | 1151.56 | 0.00 | 742.84 | 76.06 |
| ALEGEIYDTFK | 0 | 1284.62 | 5220.71 | 24087.61 | 123.88 |
| ATAVLATDIDTDSLTAFVK | 0 | 1951.02 | 1743.40 | 0.00 | 142.91 |
| DADGIVGWVK | 0 | 1058.54 | 390.12 | 1077.06 | 108.74 |
| FAESVVDGTAQAVLK | 0 | 1533.80 | 10710.20 | 23405.88 | 177.44 |
| FGVQGYPTLK | 0 | 1108.59 | 4288.19 | 28809.09 | 114.86 |
| FRMEGEFTADNVAK | 1 | 1613.75 | 6240.73 | 16914.16 | 118.52 |
| GATSPVLLGFFMEK | 0 | 1495.77 | 1389.42 | 0.00 | 113.03 |
| GFPTILFYPAGSDR | 0 | 1539.77 | 511.56 | 0.00 | 82.38 |
| IPYELPK | 0 | 858.49 | 1357.59 | 8813.89 | 91.91 |
| KVDSVIIAK | 1 | 971.60 | 1321.71 | 6532.65 | 74.92 |
| MDGTENEHPEIEVK | 0 | 1626.72 | 3140.72 | 8218.89 | 72.50 |
| MEGEFTADNVAK | 0 | 1310.58 | 1458.21 | 3334.30 | 139.86 |
| MPPTIEFNQK | 0 | 1203.60 | 429.76 | 2732.96 | 113.71 |
| NFAGEDR | 0 | 807.35 | 3889.56 | 3897.71 | 96.19 |
| NSDKIFNSGINK | 1 | 1335.68 | 5289.34 | 6597.77 | 114.76 |
| SEAIPEDPYEDGVYK | 0 | 1710.76 | 3661.35 | 10017.22 | 90.05 |
| SEAIPEDPYEDGVYKIVGK | 1 | 2108.03 | 0.00 | 2089.59 | 47.09 |
| SEKMPPTIEFNQK | 1 | 1547.77 | 2182.48 | 2179.87 | 97.46 |
| TEDVVFVQTTSADVAK | 0 | 1708.85 | 5865.27 | 14352.92 | 198.57 |
| TGPPAVTVEDADKLK | 1 | 1539.81 | 20811.50 | 35699.32 | 165.78 |
| TNAKIPYELPK | 1 | 1272.71 | 302.41 | 575.84 | 119.39 |
| TPIVFEGGDR | 0 | 1089.55 | 2422.31 | 6925.80 | 109.29 |
| TVESVVLDETK | 0 | 1218.63 | 0.00 | 7110.18 | 155.15 |
| VDATQEESLAQK | 0 | 1317.64 | 6541.44 | 19835.97 | 252.14 |
| VDSVIIAK | 0 | 843.51 | 0.00 | 998.99 | 68.92 |
|  |  |  | 119608.9 | 297738.6 |  |
